# Morpholino Analogues of Fingolimod as Novel and Selective S1P_1_ Ligands with In Vivo Efficacy in a Mouse Model of Experimental Antigen-Induced Encephalomyelitis

**DOI:** 10.3390/ijms21186463

**Published:** 2020-09-04

**Authors:** Bisera Stepanovska, Aleksandra Zivkovic, Gaby Enzmann, Silvia Tietz, Thomas Homann, Burkhard Kleuser, Britta Engelhardt, Holger Stark, Andrea Huwiler

**Affiliations:** 1 Institute of Pharmacology, University of Bern, Inselspital INO-F, CH-3010 Bern, Switzerland; bisera.stepanovska@pki.unibe.ch; 2 Institute of Pharmaceutical and Medicinal Chemistry, Heinrich Heine University Düsseldorf, Universitaetsstr. 1, D-40225 Duesseldorf, Germany; aleksandra.zivkovic@hhu.de (A.Z.); stark@hhu.de (H.S.); 3 Theodor Kocher Institute, University of Bern, Freiestrasse 1, CH-3012 Bern, Switzerland; gaby.enzmann@tki.unibe.ch (G.E.); silvia.tietz@dbmr.unibe.ch (S.T.); britta.engelhardt@tki.unibe.ch (B.E.); 4 Institute of Nutritional Science, University of Potsdam, Arthur-Scheunert Allee 114–116, D-14558 Nuthetal, Germany; homann@uni-potsdam.de (T.H.); kleuser@uni-potsdam.de (B.K.)

**Keywords:** ST-1893, ST-1894, morpholino analogues of fingolimod, sphingosine 1-phosphate, immunomodulator, lymphopenia, multiple sclerosis, experimental antigen-induced encephalomyelitis

## Abstract

Multiple sclerosis (MS) is a chronic, inflammatory, autoimmune disease of the central nervous system (CNS) which is associated with lower life expectancy and disability. The experimental antigen-induced encephalomyelitis (EAE) in mice is a useful animal model of MS, which allows exploring the etiopathogenetic mechanisms and testing novel potential therapeutic drugs. A new therapeutic paradigm for the treatment of MS was introduced in 2010 through the sphingosine 1-phosphate (S1P) analogue fingolimod (FTY720, Gilenya^®^), which acts as a functional S1P_1_ antagonist on T lymphocytes to deplete these cells from the blood. In this study, we synthesized two novel structures, ST-1893 and ST-1894, which are derived from fingolimod and chemically feature a morpholine ring in the polar head group. These compounds showed a selective S1P_1_ activation profile and a sustained S1P_1_ internalization in cultures of S1P_1_-overexpressing Chinese hamster ovary (CHO)-K1 cells, consistent with a functional antagonism. In vivo, both compounds induced a profound lymphopenia in mice. Finally, these substances showed efficacy in the EAE model, where they reduced clinical symptoms of the disease, and, on the molecular level, they reduced the T-cell infiltration and several inflammatory mediators in the brain and spinal cord. In summary, these data suggest that S1P_1_-selective compounds may have an advantage over fingolimod and siponimod, not only in MS but also in other autoimmune diseases.

## 1. Introduction

Multiple sclerosis (MS) is a chronic, inflammatory, demyelinating, autoimmune disease of the central nervous system (CNS), which affects more than 2.3 million people worldwide, mostly young adults. The pathological features of MS include the formation of plaques (lesions) in the brain and spinal cord, where inflammation, demyelination, gliosis (scarring), axonal injury, and axonal loss occur [1]. Although it is not considered as a fatal disease, it is associated with lower life expectancy and disability that affects the quality of life [2]. A new therapeutic paradigm for the treatment of MS was introduced in 2010 and featured a modulation of the sphingosine 1-phosphate receptor 1 (S1P_1_) signaling by using fingolimod (FTY720, Gilenya^®^). Fingolimod desensitizes S1P_1_ in T cells, blocking their egress from lymph nodes and leading to lymphopenia [3]. After the approval of fingolimod, which is a non-selective S1PR modulator, the search for structures with better selectivity, potency, pharmacokinetic properties, effectiveness for other autoimmune and inflammatory diseases, and fewer adverse effects was boosted [4]. At the beginning of 2020, two additional drugs, siponimod (BAF312, Mayzent^®^) and ozanimod (RPC-1063, Zeposia^®^), were approved by the European Medicines Agency for oral treatment of patients with relapsing–remitting MS (ozanimod) or the active secondary progressive form of MS (siponimod). Interestingly, siponimod and ozanimod, compared to fingolimod, exhibit selective affinity for S1P_1_ and S1P_5_, leading to a lower risk of adverse events [5], which proves the rationale for selective targeting of the S1P receptors.

The five S1P receptor subtypes belong to the family of G-protein-coupled receptors (GPCRs), which are present on the cell surface where they bind with high affinity to secreted S1P and mediate an “inside-out” signaling [6]. Versatile cellular responses are elicited upon binding of the receptors with S1P as their cognate ligand, including proliferation, survival, invasion, adhesion, and migration, pertaining to the indispensability of S1P for the normal functioning of the cardiovascular, nervous, immune, and other systems [4]. S1P and S1P receptors also regulate pathophysiological processes, including inflammatory mediator synthesis, tissue remodeling, and angiogenesis, contributing to the pathogenesis of various diseases; thus, they may serve as targets for prevention or treatment [4].

S1P_1_, S1P_2_, and S1P_3_ are ubiquitously expressed, while S1P_4_ and S1P_5_ have a restricted expression in the immune compartments and in the CNS, respectively. Lack of S1P_2/3/4_ or S1P_5_ in mice does not produce an apparent phenotype; however, lack of S1P_1_ in mice results in embryonic lethality due to an inefficient process of vascular maturation and consequent hemorrhage, pointing to a non-redundant role of S1P_1_ in the vascular system [7]. S1P_1_ also plays a significant physiological role as a key regulator of lymphocyte trafficking, and the main pharmacodynamic effect of fingolimod and siponimod lays in their ability to induce a persistent internalization of S1P_1_ in lymphocytes and subsequent lymphopenia [8].

Critical for the lymphocyte trafficking is a S1P gradient, which exists among the plasma, the interstitial space of secondary lymphoid tissues, and the lymph, and which is sensed by the lymphocytes through S1P_1_ [9]. As they move from spaces with high to low S1P concentrations, their desensitized S1P_1_ is recycled back to the cell surface and can become again responsive to S1P [10]. Fingolimod and siponimod also induce S1P_1_ internalization; however, in contrast to S1P, the receptor is irreversibly degraded in the proteasome and does not recycle back to the cell surface which reduces its membrane expression, a phenomenon known as “functional antagonism” [11]. That way, the egress of lymphocytes, particularly naïve and central memory T cells, is hindered, while effector memory T cells are spared, resulting in an overall preferential reduction of the pathogenic immune responses and sparing large parts of protective immunity in multiple sclerosis patients [12]. As autoaggressive T cells are involved in the pathology of many autoimmune diseases in addition to MS, such as Crohn’s disease, ulcerative colitis, and rheumatoid arthritis, a wider therapeutic use for S1PR modulators may be envisioned.

Apart from S1P_1_, fingolimod also targets S1P_3_, S1P_4_, and S1P_5_. While S1P_5_ is thought to mediate beneficial effects in the CNS, S1P_3_ is rather involved in adverse reactions’ signaling [4], such as vasoconstrictive events and hypertension [13]. The role of S1P_4_ in physiological processes is still poorly understood and, therefore, the effects of fingolimod mediated by S1P_4_ are vague. This highlights the need for developing compounds with a narrow S1PR selectivity profile which will allow tackling a specific pathology while having a more predictable spectrum of adverse reactions.

**Figure 1:**
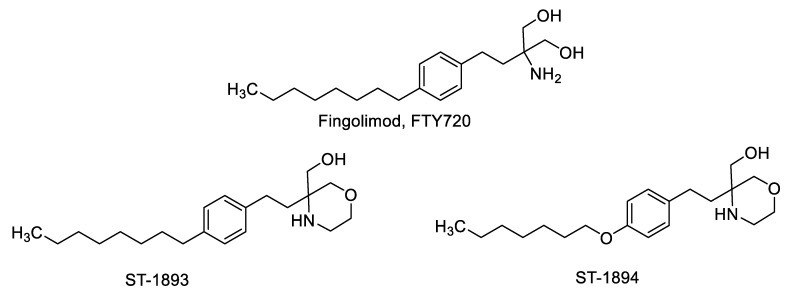
Chemical structures of fingolimod (FTY720), ST-1893, and ST-1894.

In this study, we present two novel compounds derived from fingolimod, ST-1893 and ST-1894, which chemically feature slight alterations in the polar head group by introducing a substituted morpholino ring (Figure 1), and this results in a selectivity toward S1P_1_. Both compounds were full agonists at S1P_1_, although less potent than S1P, and they also exhibited functional antagonistic activity as shown by a sustained internalization in a cell culture of S1P_1_-overexpressing Chinese hamster ovary (CHO)-K1 cells. In vivo, they exerted a lymphopenic effect in mice and were efficacious in an experimental antigen-induced encephalomyelitis (EAE) model, where they reduced the motor deficits, improved the body weight, and prevented the development of disease with the highest clinical score. Due to their S1P_1_-selective nature, they possibly exhibit fewer adverse effects and have an advantage over fingolimod and siponimod, not only in MS but also in other autoimmune diseases.

## 2. Results

**Figure 2:**
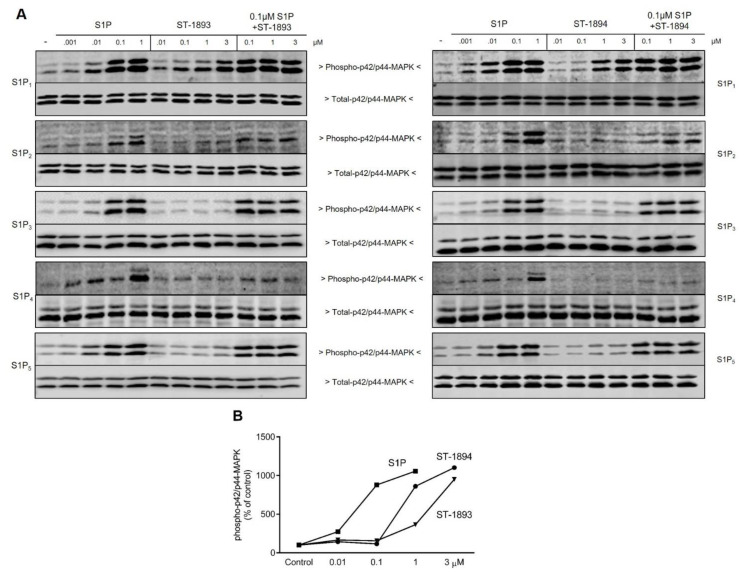
Characterization of ST-1893 and ST-1894 for agonistic and antagonistic activity on each sphingosine 1-phosphate receptor (S1PR) subtype. Starved confluent Chinese hamster ovary (CHO)-K1 cells, which stably overexpress S1P_1_, S1P_2_, S1P_3_, S1P_4_, or S1P_5_ receptors, were treated for 10 min with increasing concentrations of S1P, ST-1893 (**left panels**), and ST-1894 (**right panels**) (all in µM), or with 100 nM S1P in the presence of increasing concentrations of ST-1893 or ST-1894. Protein extracts were prepared, separated by SDS-PAGE, transferred to nitrocellulose, and subjected to Western blot analysis as described in Section 4. Membranes were incubated with phospho- and total p42/p44-mitogen-activated protein kinase (MAPK). Blots in (**A**) show one representative out of three independent experiments. The graph in (**B**) shows the quantification of p42/p44-MAPK activation in CHO-S1P_1_ cells and depicts the means of three independent experiments.

To evaluate the S1P receptor subtype activation profile of ST-1893 and ST-1894, CHO-K1 cells, that stably overexpress the different S1P receptor subtypes, were used, and phosphorylation of p42/p44- mitogen-activated protein kinase (MAPK) was measured as an early read-out of receptor activation [14,15]. Ten minutes of stimulation with ST-1893 or ST-1894 concentration-dependently increased phospho-p42/p44-MAPK in CHO-S1P_1_, while none of the other receptors (S1P_2, 3, 4_ or S1P_5_) were activated (Figure 2A).

**Figure 3:**
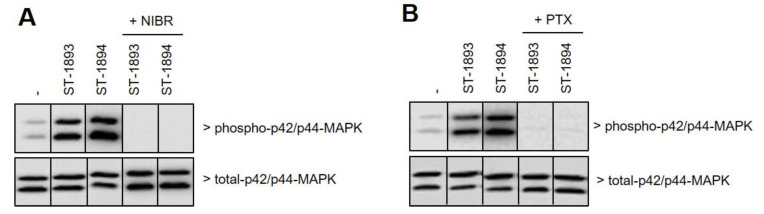
Effect of ST-1893 and ST-1894 on signaling downstream of S1P_1_. Starved confluent CHO-S1P_1_ cells were pretreated for 15 min with NIBR-0213 (NIBR, 1 μM) (**A**) or for 18 h with pertussis toxin (PTX, 100 ng/mL) (**B**) prior to stimulation for 10 min with either vehicle (dimethyl sulfoxide (DMSO)) ST-1893, or ST-1894 (both at 3 μM). Protein extracts were prepared, separated by SDS-PAGE, transferred to nitrocellulose, and subjected to Western blot analysis as described in Section 4. Membranes were probed with phospho- and total-p42/p44-MAPK, and representative samples out of three independent experiments are shown.

We further studied whether ST-1893 and ST-1894 could act as antagonists. For this, cells were stimulated with a fixed concentration of S1P in the presence of increasing concentrations of either ST-1893 or ST-1894. However, no reduction of S1P-stimulated p42/p44-MAPK activation was detected, excluding a direct antagonistic effect of ST-1893 and ST-1894 (Figure 2A). Furthermore, ST-1893- and ST-1894-stimulated p42/p44-MAPK activation was completely abolished by pretreatment with the competitive S1P_1_ antagonist NIBR-0213 (Figure 3A) [16] and by pertussis toxin (Figure 3B), thus confirming the reported signaling of S1P_1_ and the involvement of G_i/o_.

**Figure 4:**
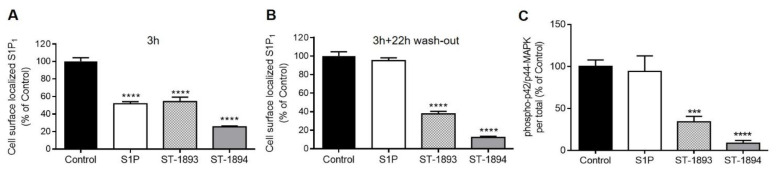
Effect of ST-1893 and ST-1894 on S1P_1_ receptor internalization and sustained S1P_1_ signaling desensitization. (**A**, **B**) Starved confluent CHO-K1 cells overexpressing N-Myc-tagged S1P_1_ receptor in 24-well plates were stimulated with equimolar concentrations (3 µM) of S1P, ST-1893, ST-1894, or DMSO control for 3 h (**A**), or 3 h followed by 22 h in serum-free conditions for recovery (**B**). Cells were fixed and further processed as explained in Section 4. Results are expressed as a percentage of control and are means ± SD (*n* = 3); **** *p* < 0.0001 compared to DMSO-treated control samples. (**C**) Starved confluent CHO-S1P_1_ cells were stimulated with 3 μM S1P, ST-1893, ST-1894, or DMSO (control) for 3 h followed by 22 h washout in serum-free conditions, and a re-stimulation for 10 min with 3 μM S1P. Cell lysates were subjected to Western blot analysis and membranes were probed with phospho- and total p42/p44-MAPK antibodies. Results are expressed as a percentage of control cells and are depicted as means ± SD (*n* = 3); *** *p* < 0.001, **** *p* < 0.0001.

As lymphocytes traffic between the circulation and secondary lymphoid tissues, they encounter substantially different S1P concentrations, which are very high in the blood and low in the tissue. High concentrations of S1P are supposed to induce desensitization of S1P_1_, and, as the concentration of S1P drops, S1P_1_ is recycled back to the cell surface and becomes again sensitive to S1P [10]. The initial phase of fingolimod-induced internalization resembles that of S1P; however, the subsequent mechanism is unique for fingolimod as it does not dissociate from the receptor, and the whole fingolimod–S1P_1_ receptor complex is sorted for degradation, resulting in receptor depletion on the surface [17]. Since fingolimod is known to induce sustained internalization and, thus, desensitization of S1P_1_, described as functional antagonism, we further tested whether ST-1893 and ST-1894 exert the same functional antagonism. To this end, an in situ ELISA was established to detect cell surface-expressed Myc-tagged S1P_1_. Stimulation of CHO-S1P_1_ cells for 3 h, with either S1P or the ST compounds, resulted in a 50–75% reduction of cell surface S1P_1_ (Figure 4A). When cells were stimulated for 3 h and a subsequent washout period of 22 h, a complete recovery of S1P_1_ in the S1P-stimulated group was observed, pointing to an efficient recycling process (Figure 4B). Conversely, in ST-1893- or ST-1894-treated samples, cell surface S1P_1_ remained low, suggesting a persistent internalization.

To further prove that sustained S1P_1_ internalization also leads to a sustained downregulation of S1P_1_ signaling, after the washout period, cells were re-stimulated with a new pulse of S1P for 10 min and taken for MAPK activation. Data showed that prolonged pre-treatment with ST-1893 or ST-1894, but not S1P, caused a downregulation of S1P signaling. As seen in Figure 4C, S1P_1_ is similarly activated in the S1P-treated group as in the control, suggesting that, within the 22 h of recovery period, S1P_1_ was recycled to the cell surface and could be stimulated again by S1P. On the other hand, due to the long-term internalization and desensitization, without receptor recycling, ST-1893 and ST-1894 groups had decreased p42/p44-MAPK activation after a new pulse of S1P stimulation.

In a further step to analyze the binding of ST compounds to the ligand-binding pocket of S1P_1_ and to determine the possibly interacting amino acids, a homologous S1P_1_ model was developed on the basis of the X-ray structure [18]. To generate an activated S1P_1_ model, the adrenergic receptor was replaced by the homologous S1P_1_ using an established β2-adrenergic receptor–arrestin complex (Figure S1, Supplementary Materials). This approach allows the development of homologous models of activated GPCR proteins [19,20]. The ligands were charged by applying the molecular operating environment (MOE) program and docking experiments were performed. Indeed, both molecules, ST-1893 (Figure S2A, Supplementary Materials) and ST-1894 (Figure S2B, Supplementary Materials) fitted well into the binding pocket as shown in the two- (Figure S2, Supplementary Materials) and the three-dimensional models (Figure S3, Supplementary Materials). Moreover, the docking results and energy minimization calculations predicted an interaction with S1P_1_ (Table S1, Supplementary Materials). The morpholino-group of ST-1893 and ST-1894 and their hydroxymethyl substituents interacted with Lys-34 (H-donor), Tyr-29 (H-donor), Glu-121 (H-acceptor), and Glu-294 (H-acceptor). These interactions with specific amino-acid residues suggested that both ST-1893 and ST-1894 may exert S1P_1_ agonist activity. The docking score for ST-1893 corresponds approximately to ST-1071 [15]; however, due to the larger volume of ST-1893, we predicted that dissociation from the receptor would be more hampered, leading to a higher activity.

Interestingly, docking experiments and calculation of binding energies indicated that a more favorable interaction with S1P_1_ occurs when both substances, ST-1893 and ST-1894, are phosphorylated (Table S1, Supplementary Materials). The binding energy of ST-1893 increased from −112.80 kcal/mol of the non-phosphorylated form to −150.15 kcal/mol when the molecule was phosphorylated. In analogy, ST-1894 shows an enhanced binding energy of the phosphorylated molecule as there was an increase from −128.09 kcal/mol to −156.47 kcal/mol. FTY720-phosphate showed in the same docking experiments a binding energy of −141.98 kcal/mol. The oxygen and a carbon from the morpholino-group from ST-1893-phosphate and ST-1894-phosphate both interacted with Asn-101 (Figure S2A,B, Supplementary Materials). The phosphorylated head of both compounds interacts with the amino acids Tyr-29 (H-donor), Lys-34 (H-donor), and Arg-120 (H-donor) similar to S1P. With the amino acid Leu-297 there is an aren-H interaction with the phenyl ring. ST-1893-phosphate interacts additionally with Phe-125 (aren-H).

**Figure 5:**
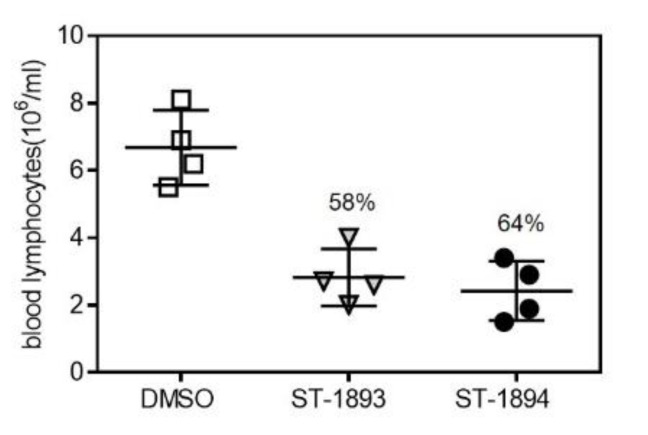
Effect of ST-1893 and ST-1894 on the number of lymphocytes in mice. C57BL/6J wild-type (wt) mice were injected intraperitoneally with 5 mg/kg ST-1893, ST-1894, or DMSO in phosphate-buffered saline (PBS) as a control, and, after 24 h, blood was collected in ethylenediaminetetraacetic acid (EDTA)-K3-coated microvettes with cardiac puncture under deep terminal isoflurane-induced anesthesia. The number of lymphocytes per mL was assessed as explained in Section 4 and is presented as means ± SD (*n* = 4).

The functional antagonism of fingolimod in vivo manifests as blocked egress from secondary lymphoid tissues and a subsequent drop in the number of lymphocytes in the blood. To confirm the functional antagonistic properties of ST-1893 and ST-1894 on S1P_1_ in vivo, wild-type C57BL/6J mice were injected intraperitoneally with 5 mg/kg of compounds or dimethyl sulfoxide (DMSO) and, after 24 h, the number of lymphocytes in blood was measured. Results show that, at the dose of 5 mg/kg of ST-1893 or ST-1894, a drop in blood lymphocytes of 58% and 64%, respectively, occurred, confirming their immunosuppressive potential (Figure 5).

**Figure 6:**
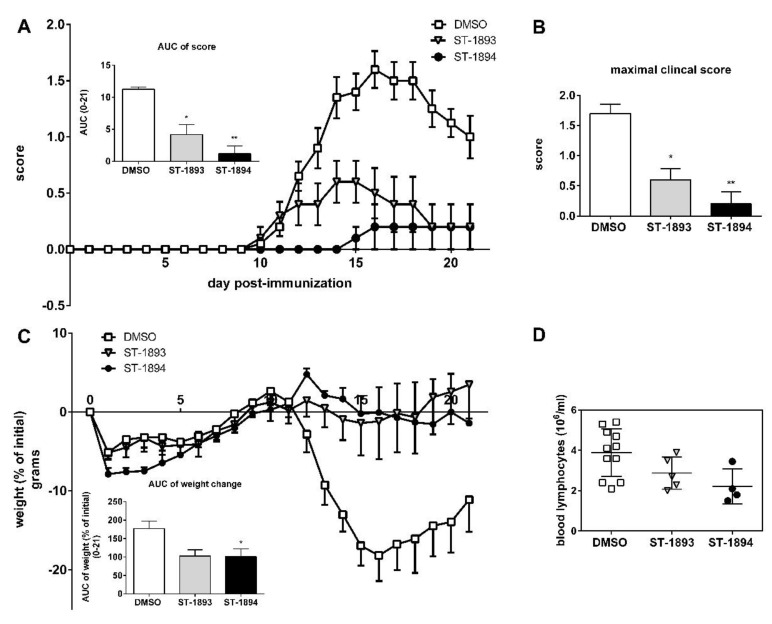
Effect of ST-1893 and ST-1894 on experimental antigen-induced encephalomyelitis (EAE) disease course. EAE was induced in C57BL/6J wt mice and, starting from day 5 until day 20 post immunization, they were injected with ST-1893, ST-1894, or DMSO dissolved in PBS at 5 mg/kg, as explained in Section 4. Graph in (**A**) represents the daily clinical score based on a four-point scoring system, and the inset graph presents the area under the curve (AUC) value from the scores from day 0 until day 21. The maximal clinical score reached for each individual mouse is presented in graph (**B**). Graph in (**C**) shows the weight change based on the weight at day 0 set as 0%, with the respective AUC calculation in inset. Results (**A**–**C**) are presented as means ± standard errors of the mean (SEM); * *p* < 0.05, ** *p* < 0.01, statistically significant when compared to the DMSO control EAE group (*n* = 10, DMSO; *n* = 5, ST-1893; *n* = 5, ST-1894). (**D**) Blood was collected at day 21 in EDTA-K3-coated microvettes with cardiac puncture, and the number of lymphocytes per mL was measured as explained above and is presented as means ± SD (*n* = 11, DMSO; *n* = 5, ST-1893; *n* = 4, ST-1894).

In a next step, to evaluate the efficacy of ST-1893 and ST-1894 in a model of autoimmune inflammatory disease, we conducted an active myelin oligodendrocyte glycoprotein (MOG)-induced EAE experiment in C57BL/6J mice, in which ST-1893 and ST-1894 were given prophylactically and daily starting at day 5 post immunization. In vehicle-treated control mice, the first motor symptom, i.e., a limp tail, appeared at approximately day 10, and, as the disease progressed, the motor signs in the vehicle-treated group aggravated and mice acquired hind leg paraplegia and loss of lower body control. ST-1893- and ST-1894-treated mice showed a much milder clinical picture, with reduced motor deficits (Figure 6A), the highest reached clinical score (Figure 6B), and improved body weight (Figure 6C). In these observations, there was a slight advantage of ST-1894 over ST-1893 at equal dose, which could be associated with the more potent effect on the S1P_1_ internalization and lymphocyte count. At day 21 post immunization, mice were sacrificed, and lymphocyte analysis in blood showed only a pattern of decrease in the ST-1893- and ST-1894-treated groups (Figure 6D), which contrasts the data obtained from healthy mice, where the depletion of blood lymphocytes was apparent and statistically significant. We attribute this discrepancy to the already low level of lymphocytes in the control group, which is in agreement with previous studies and the fact that the development of EAE itself is associated with a marked reduction in peripheral blood lymphocyte count [16,21].

**Figure 7:**
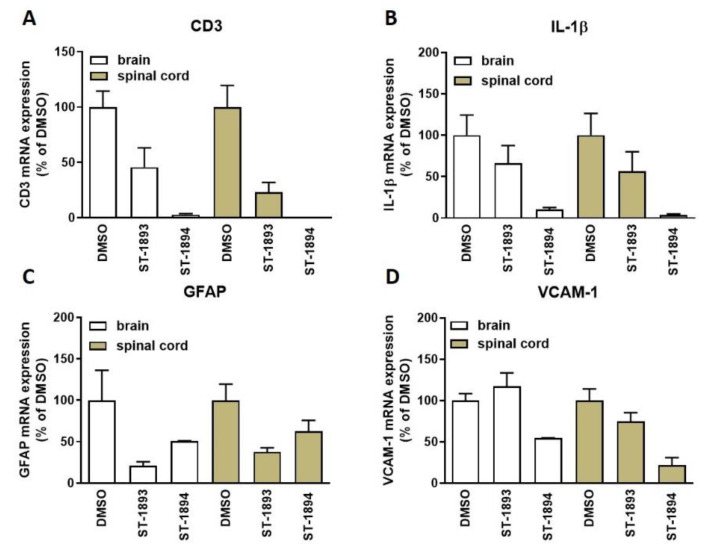
Analysis of the messenger RNA (mRNA) expression of several markers in the central nervous system (CNS) of EAE mice. CNS samples extracted from perfused EAE mice on day 21 were homogenized as explained in Section 4 and were taken for extraction of RNA. Complementary DNA (cDNA) synthesis and quantitative PCR analysis were performed, and primers for cluster of differentiation 3 (CD3) (**A**), interleukin (IL)-1β (**B**), glial fibrillary acidic protein (GFAP) (**C**), and vascular cell adhesion molecule (VCAM)-1 (**D**) were used. Results are presented as a percentage of DMSO control and are means ± SEM (*n* = 8, DMSO; *n* = 5, ST-1893; *n* = 2–3, ST-1894).

Nevertheless, tissue analysis showed a reduced expression of the T-cell marker cluster of differentiation 3 (CD3) (Figure 7A) in the CNS of ST-1893- and ST-1894-treated mice, as well as reduced messenger RNA (mRNA) expression of the inflammatory markers interleukin (IL)-1β (Figure 7B) and vascular cell adhesion molecule (VCAM)-1 (Figure 7D), as well as of the astroglial marker of scarring, glial fibrillary acidic protein (GFAP) (Figure 7C).

Since fingolimod is a prodrug and is known to strictly depend on phosphorylation by sphingosine kinase (SK)-2, but not SK-1, for therapeutic activity, we further tested whether ST-1893 and ST-1894 would also require phosphorylation by SK-2 for activity.

**Figure 8:**
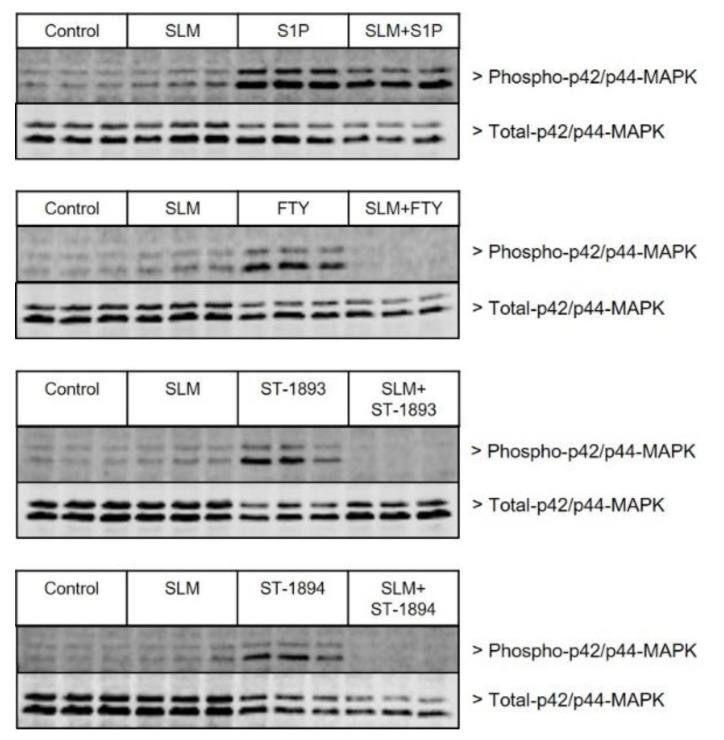
Effect of SK-2 inhibition on S1P, fingolimod, ST-1893, and ST-1894 on S1P_1_-induced p42/p44-MAPK phosphorylation. Starved confluent CHO-S1P_1_ cells were pre-stimulated for 2 h with the SK-2 inhibitor SLM6031434 (1 μM), after which they were stimulated with 100 nM S1P, 1 μM fingolimod (FTY), 1 μM ST-1893, or 1 μM ST-1894 for 10 min. Cells were harvested for usual Western blot analysis, and membranes were probed with phospho- and total-p42/p44-MAPK antibodies. Presented blots are representatives of three independent experiments.

To this end, in vitro assays were first conducted in CHO-S1P_1_ cells pretreated with the SK-2 inhibitor SLM6031434, after which they were stimulated with ST-1893 or ST-1894. S1P and fingolimod were used as positive controls for biotransformation by SK-2. Data in Figure 8 show that inhibition of SK-2 completely abolishes the activation of p42/p44-MAPK through the S1P_1_ receptor by fingolimod and confirms that preceding phosphorylation is needed for agonistic activity. In contrast, since S1P is already active, it is not affected by the inhibition of SK-2 and demonstrates no difference in the activation of S1P_1_ in the presence or absence of SLM6031434. ST-1893 and ST-1894 behaved like fingolimod and lost their S1P_1_-activating abilities in the presence of the inhibitor.

**Figure 9:**
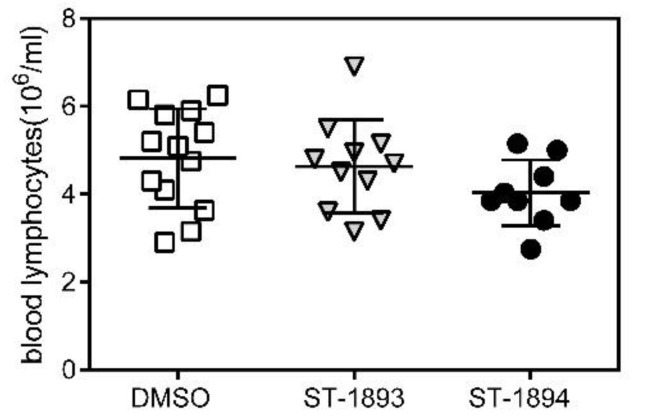
Effect of SK-2 deficiency on the lymphocyte number after ST-1893 and ST-1894 administration in vivo. C57BL/6J SK-2 knockout mice were injected intraperitoneally with 5 mg/kg ST-1893, ST-1894, or DMSO in PBS as a control, and, after 24 h blood, was collected in EDTA-K3-coated microvettes with cardiac puncture under deep terminal isoflurane-induced anesthesia. The number of lymphocytes per mL was assessed as explained in Section 4 and is presented as means ± SD (*n* = 13 for DMSO; *n* = 11 for ST-1893; *n* = 9 for ST-1894).

To investigate further the possible prodrug characteristics of ST-1893 and ST-1894, we used SK-2 knockout mice and analyzed for lymphopenia reaction after injection of ST-1893 and ST-1894. The number of lymphocytes in peripheral blood after 24 h was similar between the groups, with a tendency of decrease by ST-1894, although not significant (Figure 9). These data support the modeling study confirming that the phosphorylated structures are more potent.

**Figure 10:**
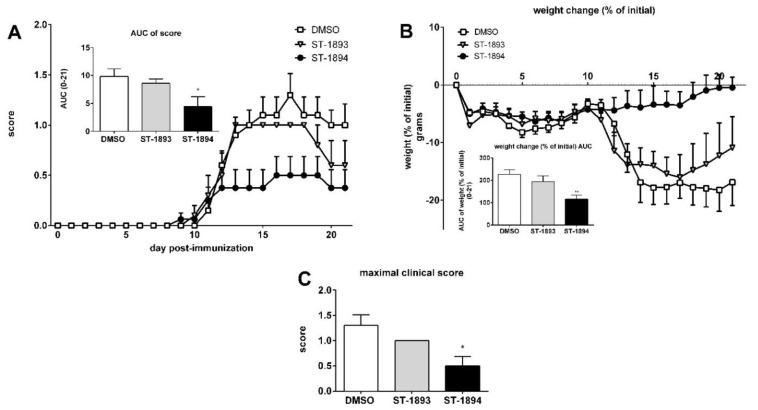
Effect of ST-1893 and ST-1894 on EAE disease course in SK-2 knockout mice. EAE was induced in C57BL/6J SK-2 knockout mice and, starting from day 5 until day 20 post immunization, they were injected with ST-1893, ST-1894, or DMSO dissolved in PBS at 5 mg/kg as explained in Section 4. Graph in (**A**) represents the daily clinical score based on a four-point scoring system, and the inset graph presents the area under the curve (AUC) value from the scores from day 0 until day 21. Graph in (**B**) shows the weight change based on the weight at day 0 set as 0%, with the respective AUC calculation in inset. The maximal clinical score reached for each individual mouse is presented in graph (**C**). Results are presented as means ± SEM (*n* = 10, DMSO; *n* = 5, ST-1893; *n* = 8, ST-1894); * *p* < 0.05, ** *p* < 0.01, statistically significant when compared to the DMSO control EAE group.

Finally, in an EAE experiment using SK-2 knockout mice, ST-1893-treated mice conferred no protection from developing the disease, while ST-1894-treated mice bore a partial protection. This was observed not only in terms of their mean daily clinical score (Figure 10A), but also in their weight (Figure 10B) and maximal clinical score (Figure 10C). Altogether, these data point to a transformation of ST-1893 and ST-1894 by SK-2 in order to reach their full immunomodulatory effectiveness in vivo.

## 3. Discussions

One decade after fingolimod gained access to the market as the first S1P receptor modulator for the treatment of multiple sclerosis, the therapeutic potential of the S1P–S1PR axis signaling took center stage in sphingolipid research. Fingolimod is an unselective modulator of four of the five S1P receptors; therefore, one of the focuses of second-generation modulators is placed on structures with narrower S1P receptor targeting, thereby achieving fewer unspecific adverse effects.

In this study, we report the synthesis and validation of two novel selective modulators of the S1P_1_ receptor, ST-1893 and ST-1894, which chemically are morpholino-derivatives of fingolimod (Figure 1). Based on their chemical structures, namely, the presence of a polar head group and a voluminous aryl chain in the *para*-position [22], we hypothesized that they would fit in the ligand-binding pocket of the S1P_1_ receptor and exert potent agonistic conformational change. Indeed, molecular modeling using a homology of S1P_1_ in its active form showed that the docking results and binding strengths fully correspond to the theory of S1P–S1P_1_ interactions (Figure S1, Supplementary Materials). On the basis of the good establishment of hydrogen bonds between the substituted morpholino-moiety on one hand and the surrounding amino-acid residues from the binding pocket on the other hand, a good S1P_1_ agonistic activity was predicted. Next, we fully characterized these compounds for agonistic and antagonistic activity on each of the S1P receptor subtypes and applied them in vivo in healthy mice with EAE as a model of MS. Both substances showed in vivo efficacy in the EAE model where they reduced the motor deficits, improved the body weight, and prevented the development of EAE with the highest clinical score (Figure 6A–C). This was accompanied by reduced T-cell infiltration in the CNS and reduced expression of several mediators which have an aggravating role in the neuroinflammatory process, which explains the ameliorated phenotype (Figure 7). In vitro, they potently activated the S1P_1_ receptor and acted as agonists at early time-points (Figure 2), while, later on, they downregulated the surface expression of the receptor (Figure 4A–C), which accounts for their lymphopenic effect in mice (Figure 5).

Our group previously characterized oxazolo–oxazolo-derivatives of fingolimod, denoted ST-968 and ST-1071, which have a dual S1P_1+3_ selectivity and turned out to be as efficient as fingolimod in vitro in cell cultures, as well as in vivo in reducing disease symptoms of EAE in mice [15]. The primary mechanism of action, for our novel morpholino-derivatives, as well as for the oxazolo–oxazolo-derivatives and fingolimod itself, relies on engaging the S1P_1_ receptor, thereby inducing lymphocyte sequestration [3]. On the other hand, it is believed that the stimulation of S1P_3_, which is ubiquitously expressed throughout the body, may be associated with some adverse reactions from fingolimod medication, such as headache, hypertension, stroke [23], cough, dyspnea, and macular edema [24]. On the other hand, the physiological role of S1P_4_, which is expressed in the immune and hematopoietic compartments, is still poorly understood and, therefore, the effects of fingolimod mediated by S1P_4_, whether beneficial or detrimental, are vague. Furthermore, the net effect of the drug binding is dependent on many factors, which include the S1P receptor repertoire and predominant expression of a particular receptor in a cell, the collection of signaling pathways within a cell, variations in the expression of both S1P receptors and downstream signaling proteins, the convergence of signaling pathways initiated by different G proteins, the localization of S1P receptors on cell membrane vs. internalized/nuclearized, etc. [25,26]. All these factors bring another level of complexity to S1P receptor signaling. Obviously, the novel structures ST-1893 and ST-1894 are devoid of unspecific effects through any of the other S1P receptor subtypes and, therefore, have an additional advantage.

Although another selective S1P_1_ agonist, SEW2871, does not target other S1P receptors; it binds and activates S1P_1_ through a combination of hydrophobic and ion–dipole interactions in the binding pocket [27], rendering it less potent at downstream G-protein binding and kinase activation. This tetraaromatic compound is a weak agonist that induces S1P_1_ internalization but then allows, similar to S1P, receptor recycling instead of receptor degradation, which translates into rapidly reversible lymphopenia [28]. ST-1893 and ST-1894 establish hydrogen bonds with surrounding amino-acid residues from the receptor and likely induce a potent signal transduction which is then evidenced by a strong reduction in the number of circulating lymphocytes in vivo. Other factors include the molecular volume, which is larger for ST-1893 as compared to the oxazolo–oxazole compound ST-1071 [15]. This might hamper the dissociation from the receptor and translate into a higher activity as predicted computationally, even though both had similar docking score.

S1P_1_ antagonism is also a valid approach to achieve a block of lymphocyte egress, and, through the induction of lymphopenia, several competitive antagonists were demonstrated to be effective in EAE, collagen-induced arthritis, traumatic brain injury, and cardiac allograft rejection animal models [16,29,30,31]. However, S1P_1_ antagonism is associated with a disturbance of the endothelial barrier integrity, especially in the lungs and skin, promoting vascular leakage, edema, and chronic remodeling with functional impairments [32,33]. ST-1893 and ST-1894 had no effect on S1P-stimulated receptor activation on any of the five S1P receptors, excluding any antagonism in the presence of S1P (Figure 2A), indicating that the lymphopenic effect is solely due to a functional antagonism.

A prerequisite for the lymphocyte-depleting activity of fingolimod is the reversible and stereoselective transformation by SK-2, in order to yield the active phospho-metabolite [34]. Contrariwise, our previously described oxazolo–oxazole substances have an advantage over fingolimod, as they do not require SK-2-mediated phosphorylation in order to become active. Notably, the SK-2 inhibitor ABC294640 (Yeliva^®^) received an orphan drug designation by the Food and Drug Administration (FDA) due to its anticancer properties in several clinical trials (NCT01488513, NCT02757326, NCT02229981, NCT03377179), although some of the effects observed in in vivo studies may be attributed to off-target effects due to its low potency at SK-2 [35]. ABC294640, as well as other SK-2 inhibitors, such as SLP120701 and ROMe, are being increasingly explored in inflammatory models, giving promising anti-inflammatory results in acute and chronic models of ulcerative colitis, viral and bacterial infections, renal inflammation/fibrosis, and EAE [36,37,38,39,40]. Co-medication of fingolimod with an SK-2 inhibitor, in theory, seems like a synergistic anti-inflammatory combination, but would clearly result in therapeutic incompatibility, due to the lack of a biotransformation step for fingolimod. In this context, using SK-2 independent structures would be mandatory, and ST-968 and ST-1071 fulfill this criterion.

The novel morpholino-derivatives, ST-1893 and ST-1894, have a different dependence on SK-2 for their in vivo activity (Figure 10). ST-1893 fully depends on SK-2, and, although ST-1894 decreases the motor deficits in the absence of SK-2, the full immunomodulatory effectiveness in vivo was observed in wild-type mice where SK-2 is normally expressed. This finding points to an inherent difference in the oxazolo–oxazole and morpholine functional groups, which dictates whether the structure will become a substrate of SK-2 or not. Since the crystal structure of SK-2 is still missing, predictions of substrate binding and structure-based drug design by molecular docking studies remain vague [35].

It is also well conceivable that the ether moiety in the aliphatic chain (present in ST-1894, but not in ST-1893) may influence the prodrug properties, as we noticed that it relatively increases the S1P_1_ activation, internalization rate, and lymphopenic effect in equivalent doses. Finally, the in vivo effects depend also on other modes of biotransformation, the stability and elimination, the volume of distribution in the secondary lymphoid tissues, etc., which warrants further studies.

In summary, here, we present two novel structures derived from fingolimod, ST-1893 and ST-1894, which possess a selective S1P_1_ activation profile. Both compounds are functional antagonists that induce sustained S1P_1_ internalization in cell culture and a profound lymphopenia in mice. As they demonstrated in vivo efficacy in the EAE model, and due to their S1P_1_-selective nature, we speculate that they will exhibit fewer adverse effects and have an advantage over fingolimod and siponimod, not only in MS but also in other autoimmune diseases.

## 4. Materials and Methods

### 4.1. Chemicals and Chemical Synthesis of ST-1893 and ST-1894

**Figure 11:**
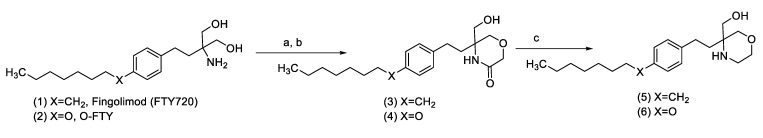
Synthesis of ST 1893 and ST-1894. (**a**) Triethylamine, chloroacetyl chloride, tetrahydrofuran (THF); (**b**) NaH, THF; (**c**) LiAlH_4_, THF.

All reagents used for synthesis are described in the Supplementary Materials. The chemical synthesis of ST-1893 and ST-1894 was done by amide and ether formation with following reduction as shown in Figure 11. Details are described in the Supplementary Materials.

### 4.2. Cell Culturing

Chinese hamster ovary (CHO)-K1 cells, which stably overexpress different human S1P_1–5_ receptor subtypes, were kindly provided by Dr. Danilo Guerini (Novartis Institutes for Biomedical Research, Basel, Switzerland). CHO-S1P_1/4/5_ cells were maintained in alpha minimum essential medium Eagle (αMEM), while CHO-S1P_2/3_ were cultivated in Roswell Park Memorial Institute (RPMI) medium. Both media were supplemented with 10% fetal bovine serum (FBS), 10 mM 4-(2-hydroxyethyl)-1-piperazineethanesulfonic acid (HEPES), 50 µg/mL gentamycin, and 0.5 mg/mL G418 as a selection antibiotic. Cells were cultured in an environment of 37 °C with 5% CO_2_ and were passaged 2–3 times a week, using Trypsin-ethylenediaminetetraacetic acid (EDTA) 0.25% for detachment. Prior to stimulation, cells were rendered serum-free overnight with a medium consisting of Dulbecco’s modified Eagle medium (DMEM), 0.1 mg/mL bovine serum albumin (BSA), and 10 mM HEPES (later on referred to as serum-free medium).

### 4.3. Western Blot Analysis

Western blot analysis was performed as previously described [41] by incubating membranes with antibodies for 16 h at 4 °C. Commercially available antibodies used were as follows: phospho-Thr^202^/Tyr^204^ p42/p44-MAPK (Cell Signaling, cat. no. 4377, 1:1000 dilution) and Myc-tag (Cell Signaling, cat. no. 2276, 1:1000 dilution). IRDye^®^ or horseradish peroxidase (HRP)-linked secondary antibodies were used, and bands were detected and analyzed by the LI-COR Image Studio^TM^ (v. 3.1; LI-COR Biotechnology GmbH, Bad Homburg, Germany). Signal intensity was evaluated by ImageJ (National Institutes of Health, USA). Polyclonal antisera against p42-MAPK and p44-MAPK were previously generated and characterized [42] and used in a dilution of 1:3000.

### 4.4. RNA Extraction and Quantitative PCR Analysis

RNA extraction, complementary DNA (cDNA) synthesis, and quantitative PCR analysis were performed as previously described [43]. Primer sequences of mouse CD3, VCAM-1, and 18S RNA were as previously described [15,44]. The sequences for other primers were as follows: GFAP, forward: CCAGCTTCGAGCCAAGGA, reverse: GAAGCTCCGCCTGGTAGACA; IL-1β, forward: TGTCCTGTGTAATGAAAGACGG, reverse: TCTTGTGACCCTGAGCGAC.

### 4.5. S1P Receptor Activation Studies

Confluent CHO-S1P_1–5_ cells were incubated in serum-free medium for 20 h and then stimulated with the indicated compounds for 10 min at 37 °C. Cell monolayers were washed with phosphate-buffered saline (PBS) and lysed in lysis buffer at 4 °C and further processed for protein separation by SDS-PAGE, transfer to nitrocellulose membranes, and Western blot analysis.

### 4.6. S1P_1_ Internalization Studies by ELISA

Confluent CHO-S1P_1_ cells in 24-well plates were starved for 20 h prior to stimulation with 3 μM of compounds in serum-free medium. In the first experimental set-up, the stimulation was ceased after 3 h, whereas, in the second set-up, the long-term effect on receptor internalization was assessed by stimulation for 3 h, washing, and placing in serum-free conditions for an additional 21 h. Cells were washed three times with ice-cold PBS, fixed with 4% paraformaldehyde in PBS at room temperature for 20 min, and blocked in 6 % (*w/v*) non-fat dry milk in PBS (below labeled as blocking buffer) for 1 h. They were incubated with anti-Myc Tag antibody (1:1000 in blocking buffer) and later on with anti-mouse HRP-linked antibody (1:5000 in blocking buffer), both for 1 h, with washing steps in between (8 min × 3 times in PBS). After 3,3′,5,5′-tetramethylbenzidine (TMB) was added as a substrate for the HRP, the reaction was stopped with 1 N H_2_SO_4_, and the absorbance was measured, subtracting the blank where the primary antibody was omitted. In order to normalize with the number of cells, we proceeded with washing, staining with 0.1% crystal violet solution in 20% methanol, and solubilizing with 5% acetic acid. Absorbance was measured, normalized with the values from the ELISA, and calculated to the DMSO control being 100%.

### 4.7. Lymphocyte Count

C57BL/6J mice were injected intraperitoneally with 5 mg/kg of ST-1893, ST-1894, or DMSO in PBS, and, after 24 h, blood was collected in EDTA-K3-coated microvettes (Sarstedt; Sevelen, Switzerland) with cardiac puncture under deep terminal isoflurane-induced anesthesia. The number of lymphocytes was assessed with a Scil Vet ABC^TM^ Hemocytometer (Scil animal care company GmbH; Viernheim, Germany).

### 4.8. Experimental Antigen-Induced Encephalomyelitis Experiments

All mice experiments were in accordance with the Swiss animal experimentation legislation and under the license number BE50/17. Active EAE was induced in C57BL/6J mice with MOG_aa35–55_ emulsified in complete Freund’s adjuvant (CFA) and pertussis toxin as described previously by our group [15]. The overall wellbeing, weight, and signs of disease were evaluated twice daily using a four-point scoring system. Disease sign scores were as follows: 0.5: limp tail, 1: hind leg weakness and unsteady gait, 2: hind leg paraplegia, 3: hind leg paraplegia, incontinence, and loss of lower body control. From day 5 until day 20 post immunization, they received daily 5 mg/kg of ST-1893, ST-1894, or DMSO in PBS via intraperitoneal injection. On day 21, blood was collected, the mice were sacrificed and perfused with ice-cold PBS, and the CNS was extracted and snap-frozen in liquid nitrogen until further analysis.

### 4.9. Organ Homogenization

The snap-frozen tissues in liquid nitrogen were homogenized using Sartorius Mikro-Dismembrator S (Göttingen, Germany) at 3000 rpm for 1 min each. The pulverized tissues were distributed in Eppendorf tubes; then, RNA-Solv^®^ reagent was added and vortexed for 5 min to aid the dissolution. Samples were centrifuged at 13,000 rpm for 10 min at 4 °C, and the supernatant was used for RNA extraction and quantitative PCR analysis as described above.

### 4.10. Molecular Modeling

Molecular docking and energy minimization experiments were performed as disclosed in the Supplementary Materials.

### 4.11. Statistical Analysis

GraphPad Prism 6 Software (San Diego, CA, USA) was used for statistical analysis and graph representations. The statistical significance was calculated with ordinary one-way ANOVA without matching or unpaired *t*-test where applicable. The level of significance was calculated with Bonferroni correction for multiple comparisons. For the EAE experiments, the nonparametric Kruskal–Wallis test for the area under the curve (AUC) was used. Data are depicted as means ± SD for *n* number of independent experiments unless otherwise stated.

## Supplementary Materials

The following are available online at https://www.mdpi.com/1422-0067/21/18/6463/s1: Figure S1. Flow of the building of the activated S1P1 model: new approach to model development; Figure S2. Two-dimensional (2D) model of interactions of ST-1893 and its phosphate (A) or ST-1894 and its phosphate (B) with amino-acid residues in the binding pocket of S1P1; Figure S3. Three-dimensional (3D) model of docking of ST-1893 and its phosphate (A) or ST-1894 and its phosphate (B) into the binding pocket of S1P1; Table S1. Interaction energies between ligand and the S1P1 receptor.

## Abbreviations

AUC	area under the curve
BSA	bovine serum albumin
CD3	cluster of differentiation 3
cDNA	complementary deoxyribonucleic acid
CFA	complete Freund’s adjuvant
CHO	Chinese hamster ovary
CNS	central nervous system
DMEM	Dulbecco’s modified Eagle medium
DMSO	Dimethyl sulfoxide
EAE	experimental antigen-induced encephalomyelitis
ECL	enhanced chemiluminescence
ELISA	enzyme-linked immunosorbent assay
FBS	fetal bovine serum
GFAP	glial fibrillary acidic protein
GPCR	G-protein-coupled receptor
HRP	horseradish peroxidase
IL-1β	interleukin 1 beta
MAPK	mitogen-activated protein kinase
αMEM	minimum essential medium Eagle, alpha modification
MOG	myelin oligodendrocyte glycoprotein
MS	multiple sclerosis
mRNA	messenger ribonucleic acid
PBS	phosphate-buffered saline
PECAM-1	platelet and endothelial cell adhesion molecule 1
PTX	pertussis toxin
S1P	sphingosine 1-phosphate
S1PR	sphingosine 1-phosphate receptor
SK	sphingosine kinase
TMB	3,3’,5,5’-tetramethylbenzidine
VCAM-1	vascular cell adhesion molecule 1
wt	wild type
