# Supplementary material for: Morpholino Analogues of Fingolimod as Novel and Selective S1P1 Ligands with In Vivo Efficacy in a Mouse Model of Experimental Antigen-Induced Encephalomyelitis
Source: Int J Mol Sci. 2020 Sep 4;21(18):6463. doi: 10.3390/ijms21186463 (PMC7555234; doi:10.3390/ijms21186463)
Supplement: Supplementary file 1 [file ijms-21-06463-s001.pdf]

Supplementary Methods and Data

Stepanovska et al.

Morpholino analogues of fingolimod as novel and selective S1P₁ ligands with *in vivo* efficacy in a mouse model of experimental antigen-induced encephalomyelitis.

Methods

Chemicals and reagents:

All basic chemicals were of highest possible purity. Roti®-Quant, DMEM with 4.5 g/L D-glucose and RPMI 1640 medium were obtained from Carl Roth GmbH (Karlsruhe, Germany). KiCqStart™ SYBR® Green qPCR ReadyMix™, cOmplete™ protease inhibitor cocktail, fatty acid- and globulin-free bovine serum albumin (BSA), BSA fraction V, dimethyl sulfoxide (DMSO), gentamycin and horse serum, were from Sigma-Aldrich Chemie GmbH (Buchs, Switzerland). RNA-Solv® reagent from Omega Bio-tek Inc., (Norcross, USA); First Strand cDNA Synthesis kit and αMEM medium from Thermo Scientific (Waltham, USA); TMB ELISA substrate (fast kinetic rate) from Abcam (Cambridge, UK). S1P was from Avanti Polar Lipids Inc. (Alabaster, USA), fingolimod from Axon Medchem (Groningen, Netherlands), NIBR-0213 from Glixx Laboratories (Hopkinton, USA), SLM6031434 from Tocris (through BioTechne AG; Zug, Switzerland). For the EAE experiments, $MOG_{aa35-55}$(MEVGWYRSPFSRVVHLYRNGK) was used from Alpha Diagnostic International (San Antonio, USA), pertussis toxin (PTX) from Research Biochemicals International (Natick, USA), complete Freund's adjuvant (CFA) from Santa Cruz Biotechnology Inc. was supplemented with 4 mg/ml desiccated *Mycobacterium tuberculosis* (H37 Ra) from BD Biosciences (Eysins, Switzerland), and isoflurane (Forene®; AbbVie AG, Baar, Switzerland). IRDye® 800CW secondary antibodies were bought from LI-COR (Lincoln, USA), and ECL™ HRP-linked antibodies, as well as ECL™ Western Blotting Detection Reagents were obtained from GE Healthcare Limited (Buckinghamshire, UK). Trypsin-EDTA 0.25% and G418 (Geneticin™) were from Life Technologies Limited (Paisley, UK). Fetal bovine serum (FBS) was purchased from PAA Laboratories (Catalogue No. A15-101, Cölbe, Germany). All oligonucleotide primers were from Eurofins Genomics GmbH (Ebersberg, Germany).

Synthesis of compounds 5 (ST-1893) and 6 (ST-1894):

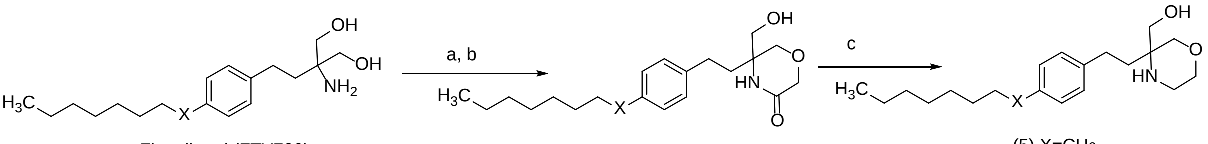

(1) X=CH2, Fingolimod (FTY720)
(2) X=O, O-FTY

(3) X=CH2
(4) X=O

(5) X=CH2
(6) X=O

a) Triethylamine, chloroacetyl chloride, THF; b) NaH, THF; c) $LiAlH_4$, THF

The chemical synthesis of the starting compounds (**1**) and (**2**) has been performed according to previously optimized procedures [1]. Compounds (**3**) and (**4**) were synthesised in one-pot two step procedure with amide formation and ring closure. The final compounds ST-1893 and ST-1894 have been obtained after reduction with complex hydrids.

All starting materials have been obtained from Sigma Aldrich and Apollo Scientific and been used without further purification. Analytical thin-layer chromatography (TLC) was carried out on precoated TLC sheets ALUGRAM® Xtra SIL G/UV254 (Macherey-Nagel, Dueren, Germany) with visualization under UV light. Mass spectra have been determined using Advion Mass Express. Atmospheric-pressure chemical ionization (APCI) was used as a method of ionization, operating in a positive mode. Data have been shown as ([M+H]+). Lyophilisation was performed on Christ Alpha 1-4 L D Plus and Alpha 2-4 LD Plus lyophilisator. ^{1}H and ^{13}C NMR spectra of final compounds were measured at Bruker Avance III - 300 (Year 2010). As solvents for NMR DMSO-d_6 and as standard tetramethylsilane (TMS) were used. Chemical shifts have given as parts per million (ppm) and been reported as s (singlet), d (doublet), dd (double of doublets), t (triplet), q(quintet) or m (multiplet), Coupling constant (J) were given in Hertz (Hz). Purification of compounds have been accomplished using flash chromatography: Biotage Isolera™ Spektra Systems with ACI™ and Assist (Biotage, Uppsala, Sweden). As a stationary phase, SNAP KP-Sil, ZIP® (Biotage, Uppsala, Sweden) was used, and as a mobile phase, dichloromethan and MeOH (Gradient 0-5%) were used. Solvents were evaporated on a Rotavapor R II (Büchi, Flawil, Switzerland) and the compounds were dried on a high-vacuum pump (Vacuubrand Chemie- Hybrid-Pumpe RC 6 (Vacuubrand, Wertheim, Germany). Compounds purity was determined by elementary analysis Vario MICRO cube Elemental Analyzer (Elementar Analysensysteme, Hanau, Germany).

*5-(Hydroxymethyl)-5-(4-octylphenethyl)morpholin-3-one (**3**)*: Fingolimod, prepared as previously reported [1] (640 mg, 2.08 mmol) was dissolved in THF (20 mL) and TEA (1.2 eq, 2.50 mmol, 350 µL) was added. The reaction mixture was cooled to 0 °C and chloroacetyl chloride (1.2 eq, 2.50 mmol, 210 µL) were added dropwise. The reaction mixture was allowed to stir at RT for 2h, evaporated to dryness and extracted with EtOAc. Organic phase was washed with $NaHCO_3$ (sat.) and water, dried with $MgSO_4$ and evaporated. Crude product was dissolved in THF (20 mL) and 3 eq (6.24 mmol, 250 mg) of 60 % NaH suspension on oil were added. After 21 h at RT additional 5 eq. of NaH were added (10.4 mmol, 416 mg). After additional 24 h at RT, the reaction was quenched with sat. NH_4Cl solution and extracted three times with EtOAc. Collected organic phases were dried over $MgSO_4$ and evaporated to dryness. Crude product was purified using flash chromatography (DCM/MeOH) to obtain 200 mg of pure product (28 %). ^{1}H NMR:δ [ppm] (300 MHz, DMSO-d_6) δ 7.96 (s, 1H), 7.09 (s, 4H), 4.99 (t, J = 5.5 Hz, 1H),

3.93 (s, 2H), 3.80 – 3.40 (m, 3H), 3.29 – 3.18 (m, 1H), 2.64 – 2.51 (m, 4H), 1.83 – 1.44 (m, 4H), 1.33 – 1.16 (m, 10H), 0.85 (t,*J*=6.6 Hz, 3H); APCI (+):348.7 ($[M+H]^+$);

5-(4-(Heptyloxy)phenethyl)-5-(hydroxymethyl)morpholin-3-one (**4**)*:* Synthesised accordingly to the procedure described for compound (**3**); Yield (28 %). ^{1}H NMR:δ [ppm] (300 MHz, DMSO-*d₆*) δ 7.95 (s, 1H), 7.09 (d, *J* = 8.5 Hz, 2H), 6.83 (d, *J*=8.5 Hz,2H), 4.98 (t, *J* = 5.6 Hz, 1H), 4.04 – 3.84 (m, 4H), 3.81 – 3.24 (m, 8H), 1.79 – 1.55 (m, 4H), 1.33 (m, 8H), 0.87 (7,*J*=7.0 Hz 3H); APCI (+):350.2 ($[M+H]^+$);

(3-(4-Octylphenethyl)morpholin-3-yl)methanol (ST-1893, **5**)*:* Compound (**3**) (180 mg, 0.52 mmol) in 20 mL of THF, was added dropwise to $LiAlH_4$ in THF (1M, 4,0 mL, 8 eq) at 0 °C. Upon addition the mixture was refluxed over night. Reaction mixture was cooled to 0°C and worked up with water and 1M NaOH solution. Stirring continued for 30 min at RT and filtrated over cellite. THF was evaporated to dryness and portioned between Et2O and water three times. Collected organic phases were washed with water and brine. Compound was precipitated from Et2O with HCl solution in *i*PrOH(5-6 M). ^{1}H NMR:δ [ppm] (300 MHz, DMSO-d_6):MHz, δ 7.18 – 7.00 (m, 4H), 4.58 (t, *J* = 5.3 Hz, 1H), 3.54 – 3.22 (m, 10H), 2.87 – 2.60 (m, 2H), 1.74 – 1.41 (m, 4H), 1.30 – 1.16 (m, 10H), 0.81 (t, *J*=7.0 Hz 3H). ^{13}C NMR (75 MHz, DMSO-d_6) δ 139.80, 136.99, 129.05, 128.53, 71.83, 66.84, 63.79, 54.14, 39.81, 35.50, 31.68, 30.75, 28.62, 27.54, 22.48, 14.03. APCI (+):334.4 ($[M+H]^+$); El.Analysis: Calculated: C (67.96%), H(9.52%), N(3.72%); Found: C(68.18 %), H (9.81%), N(3.79%);

(3-(4-(Heptyloxy)phenethyl)morpholin-3-yl)methanol (ST-1894, **6**)*: S*ynthesised accordingly to the procedure described for compound (**5**). ^{1}H NMR:δ [ppm] (300 MHz, DMSO-d_6):MHz, ^{1}H NMR (300 MHz, DMSO-d_6) δ 7.08 (d,*J*=8.5 Hz, 2H), 6.80 (d, *J*=8.5 Hz 2H), 4.57 (t, *J* = 5.3 Hz, 1H), 3.90 (t, *J* = 6.5 Hz, 2H), 3.53 – 3.22 (m, 8H), 2.81 – 2.58 (m, 2H), 1.75 – 1.46 (m, 4H), 1.32 (m, 10H), 0.81 (t,*J*=7.0 Hz 3H); ^{13}C NMR (75 MHz, DMSO-d_6) δ 157.46, 137.09, 128.93, 115.20, 71.83, 67.75, 66.84, 63.79, 54.14, 39.81, 31.72, 29.44, 29.42, 29.36, 27.54, 26.00, 22.48, 14.03. APCI (+):336.6 ($[M+H]^+$); El.Analysis: Calculated: C (64,58%), H(9.21%), N(3.77%); Found: C(64.18 %), H (8.99%), N(3.62%);

Molecular modeling experiments:

Molecular docking and energy minimization experiments were performed using the Molecular Operating Environment (MOE) molecular modeling program 2019.01 (MOE2019.01 from Chemical Computing Group Inc., 1010 Sherbooke St. West, Suite #910, Montreal, QC, Canada, H3A 2R7, 2012), Molegro 6.0 [2], Yasara 20.4.24 [3, 4] and CLC Drug Discovery Workbench Version 3.0 Qiagen Aarhus A/S.

Protein modeling of the $S1P_1$ receptor:

A homologue model of an activated $S1P_1$ receptor was developed, using the protein sequence of $S1P_1$ and corresponding templates. Templates applied for the modeling of $S1P_1$ were X-ray structures

deposited in the Protein Data Bank (PDB) under the PDB codes 3V2W and 3V2Y [5, 6]. The fusion protein T4L of the X-ray structure [7] was removed and the resulting fragmentary $S1P_1$ protein was used as template to generate a full-length $S1P_1$ protein via the Fasta sequence (UniProt KB - P21453). The structures were prepared for further modeling by adding hydrogen atoms and partial charges to the peptides via the Protonate3D application of MOE2019.01 (Chemical Computing Group ULC, 1010 Sherbrooke St. West, Suite #910, Montreal, QC, Canada, H3A 2R7). This process was repeated for each model as an intermediate step. Then, the ligand ML05 was removed and S1P was placed in the active center by means of docking examinations (pharmacophore controlled).

In Yasara, the homology modeling macro in the standard configuration was utilized. The consensus models were then built with MOE and Yasara by applying the homology macro. The missing chains were supplemented and optimized by intermittent side chain constructions. The missing tertiary structures of the protein sequence were calculated and completed in steps of 5 amino acids each. The consensus homologue model was the result of 500 homologue structures optimized by molecular dynamics (MD) simulation with Yasara 20.4.24. [8-10].

The model with the ligand S1P was also examined by MD simulation. The stereochemistry quality aspects of the resulting model were checked using MOE (Ramachandran plot). To generate an activated $S1P_1$ homology model, the intermediate model was fused with Arrestin and the corresponding complex was subjected to a comprehensive MD investigation. Thus, structural stability of the overall model could be achieved. From these, the $S1P_1$ receptor was separated and used as a novel homologous model for docking studies.

Ligand structure optimization and docking:

The initial structures of the ST-1893, FTY720-phosphate and ST-1894 were sketched with Chemdraw Suite 2016 and transferred to a MOE database. The phosphorylated compounds were designed with MOE as 3D structures. All structures were minimized until a root mean square deviation (RMSD) of 0.01 kcal/mol Å was reached. The Energy minimization was performed using the MMFF94 force field option [11] with the restriction to preserve original chirality of the molecules. In Molegro, the docking algorithm called MolDock was used to find the correct poses [2].

No usable results were found with the plant algorithm. The further development of Molegro Plant in the CLC Drug Discovery Workbench 3 (test license) was also used, but here too no valid results could be obtained. In MOE we used the algorithm MOE Dock. The docking protocols for rigid receptor and induced fit were used.

Molecular dynamics (MD):

The best complex for each ligand and receptor was then subjected to MD simulations (Refinement) using Amber 14. A simulation cell was constructed around the models (2 x 7.5 Å larger than the model) with a 7.9 Å real space cut-off for the electrostatic force calculated used the Particle Mesh Ewald method. The pKa values of the ionizable groups were predicted and assigned protonation states based on pH 7.4 (temperature= 298K, density=0.997). The cell was filled with water and the Yamber electrostatic potential was evaluated at all water molecules, the molecules with the lowest or highest potential were turned into sodium or chloride counter ion until the cell was neutral. A short steepest descent minimization was performed to remove severe bumps followed by simulated annealing minimizations at 298 K. Then, MD simulations were run with Amber 14 force field at 298 K and 0.9% NaCl in the simulation cell for 500 ps to refine the models. For further analysis simulation snapshots were captured every 25 ps. For the intermediate and final models, the MD simulation was carried out over a period of 100 ns.

Results:

Ligand	E-Inter (protein – ligand)
S1P	-169,07
FTY720-P	-141,98
ST1893	-112,80
ST1893-P	-150,15
ST1894	-128,09
ST1894-P	-156,47

Table S1: Interaction energies between ligand and the $S1P_1$ receptor.

Interactions energies were calculated by Molegro as described in the supplementary Methods Section. E-inter (protein-ligand) indicates the energy released upon ligand binding and is indicated as kcal/mol.

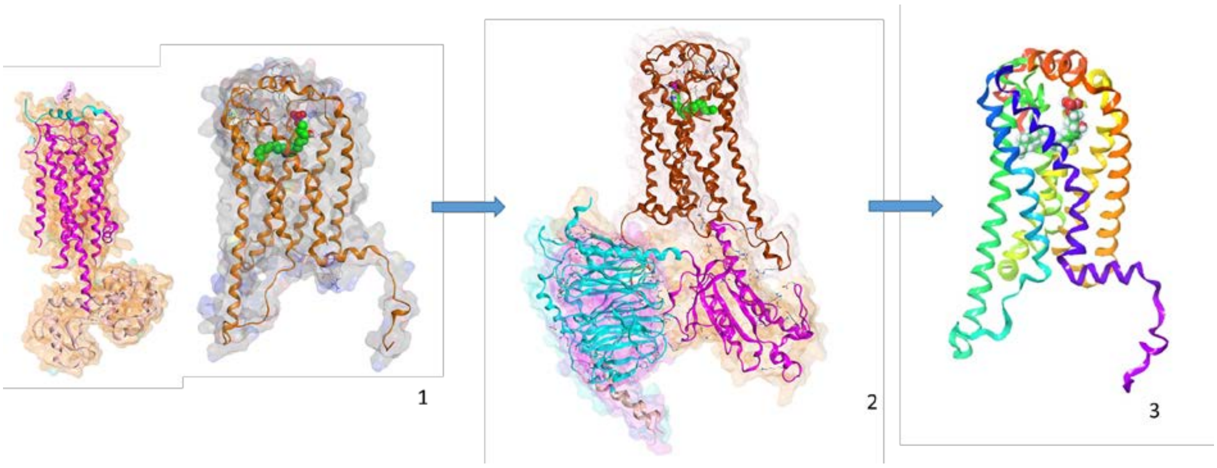

S1A

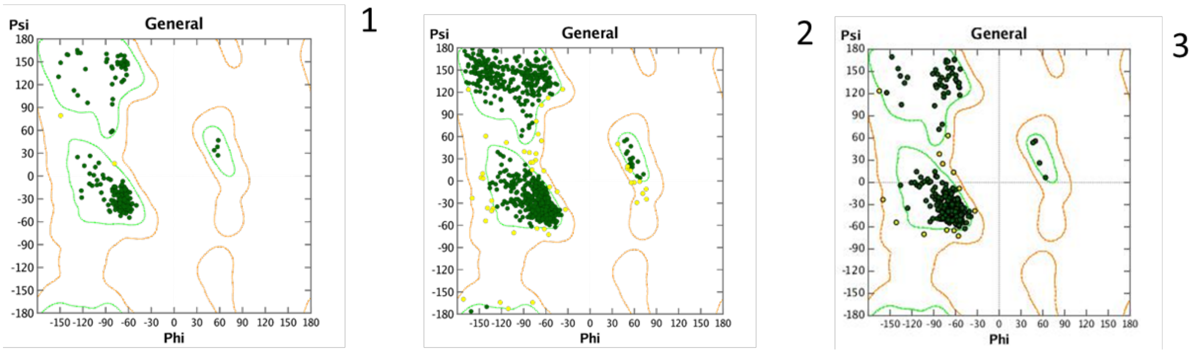

S1B

Fig. S1: Flow of the building of the activated $S1P_1$ model: New approach to model development.
(A) After the development of the Standard Model (1), it is fused with the binding protein arrestin (2). This complex is optimized by MD studies until it leads to a stable valid structure. From this complex the S1P1 is cut out and the final model is used (3).
(B) Ramachandran plot for the quality control of the models.

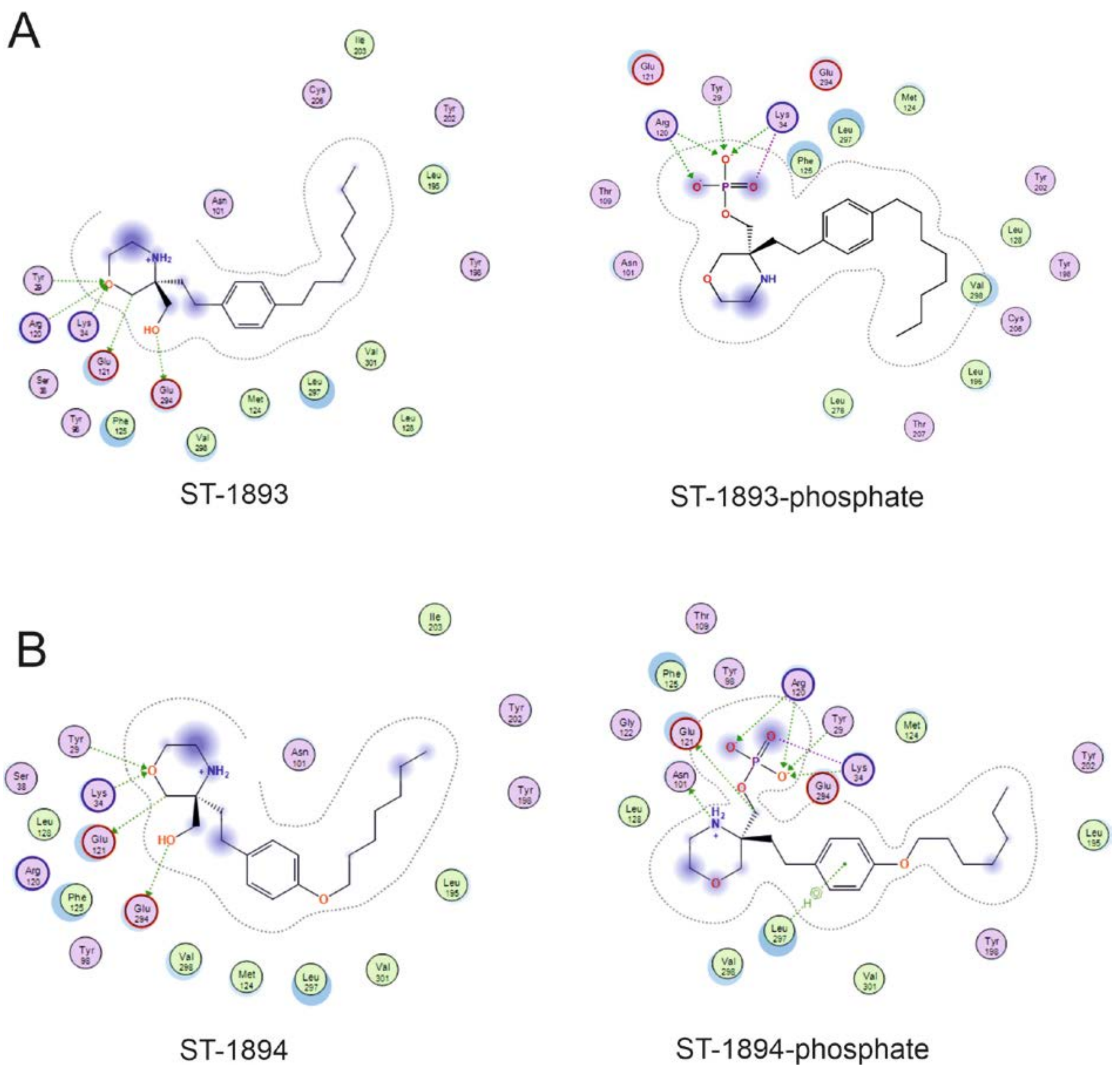

Fig. S2: 2D-model of interactions of ST-1893 and its phosphate (A) or ST-1894 and its phosphate (B) with amino acid residues in the binding pocket of S1P$_1$.

Molecular docking experiments were performed using the Molecular Operating Environment (MOE) modeling program as explained in the Supplementary Methods Section. Polar amino acids from the receptor's binding pocket are colored in pink and hydrophobic in green; acidic amino acids have additional red contour ring and the basic have a blue contour ring. The degree of interaction with atoms from the ligand is presented by a light blue halo around the amino acid, where a larger halo means a stronger interaction. Established hydrogen bonds are depicted by green arrows, while blue clouds on the ST-compounds' atoms indicate the surface area exposed to the solvent.

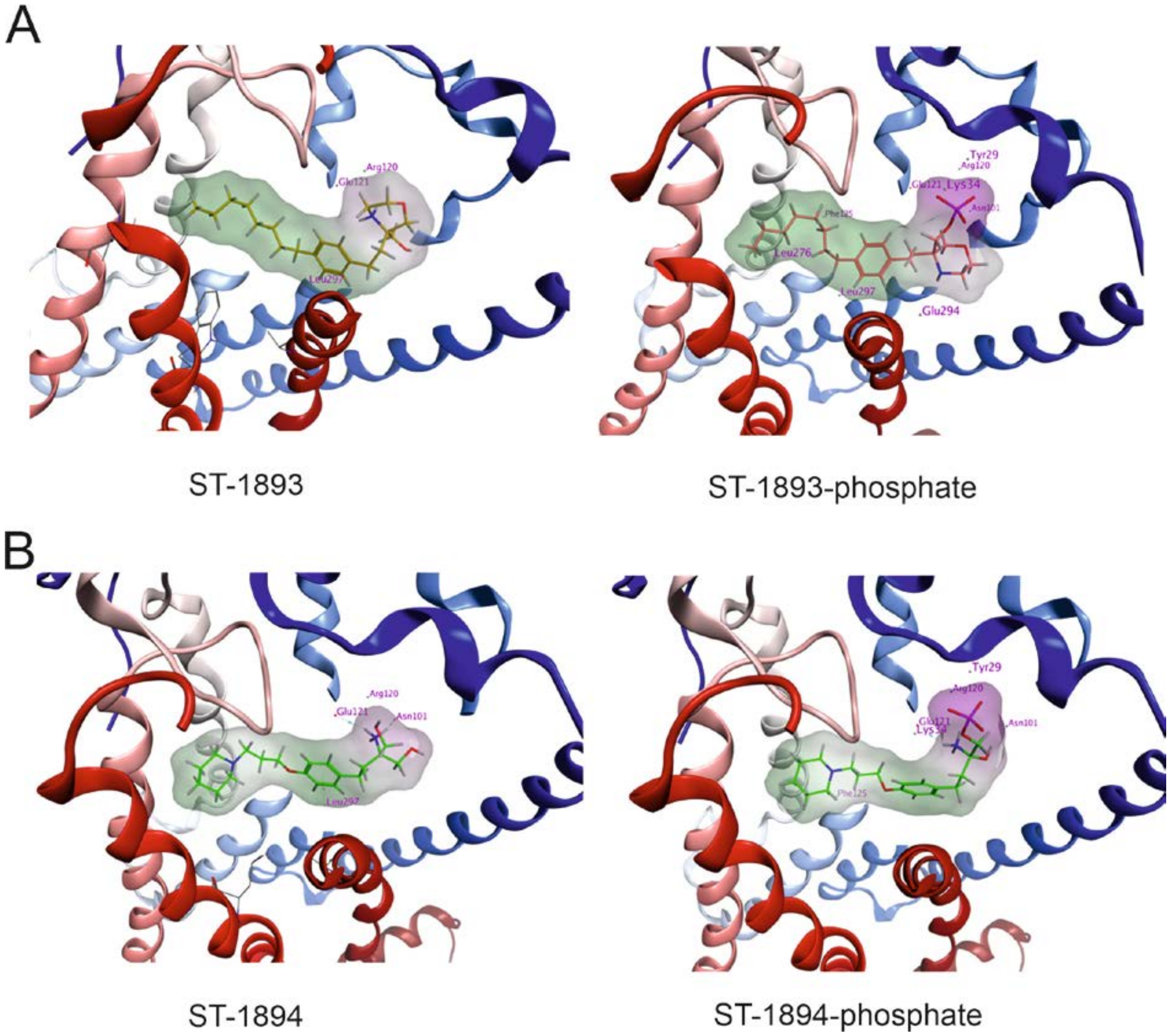

Fig. S3: 3D-model of docking of ST-1893 and its phosphate (A) or ST-1894 and its phosphate (B) into the binding pocket of S1P$_1$.

References:

1. Zivkovic, A.; Stark, H., Efficient chromatography-free synthesis of the oxy-analogue of fingolimod. *Tetrahedron Lett* **2010,** 51, (29), 3769-3771.
2. Thomsen, R.; Christensen, M. H., MolDock: a new technique for high-accuracy molecular docking. *Journal of Medicinal Chemistry* **2006,** 49, (11), 3315-21.
3. Krieger, E.; Koraimann, G.; Vriend, G., Increasing the precision of comparative models with YASARA NOVA--a self-parameterizing force field. *Proteins* **2002,** 47, (3), 393-402.
4. Krieger, E.; Joo, K.; Lee, J.; Raman, S.; Thompson, J.; Tyka, M.; Baker, D.; Karplus, K., Improving physical realism, stereochemistry, and side-chain accuracy in homology modeling: Four approaches that performed well in CASP8. *Proteins* **2009,** 77 Suppl 9, 114-22.
5. Berman, H. M.; Westbrook, J.; Feng, Z.; Gilliland, G.; Bhat, T. N.; Weissig, H.; Shindyalov, I. N.; Bourne, P. E., The protein data bank. *Nucleic acids research* **2000,** 28, (1), 235-242.
6. Hanson, M. A.; Roth, C. B.; Jo, E.; Griffith, M. T.; Scott, F. L.; Reinhart, G.; Desale, H.; Clemons, B.; Cahalan, S. M.; Schuerer, S. C., Crystal structure of a lipid G protein–coupled receptor. *Science* **2012,** 335, (6070), 851-855.

7. Chun, E.; Thompson, A. A.; Liu, W.; Roth, C. B.; Griffith, M. T.; Katritch, V.; Kunken, J.; Xu, F.; Cherezov, V.; Hanson, M. A., Fusion partner toolchest for the stabilization and crystallization of G protein-coupled receptors. *Structure* **2012,** 20, (6), 967-976.
8. Chinea, G.; Padron, G.; Hooft, R.; Sander, C.; Vriend, G., The use of position-specific rotamers in model building by homology. *Proteins: Structure, Function, and Bioinformatics* **1995,** 23, (3), 415-421.
9. Dunbrack Jr, R. L.; Cohen, F. E., Bayesian statistical analysis of protein side-chain rotamer preferences. *Protein Science* **1997,** 6, (8), 1661-1681.
10. Canutescu, A. A.; Shelenkov, A. A.; Dunbrack Jr, R. L., A graph-theory algorithm for rapid protein side-chain prediction. *Protein science* **2003,** 12, (9), 2001-2014.
11. Halgren, T. A., Merck molecular force field. I. Basis, form, scope, parameterization, and performance of MMFF94. *J Comput Chem* **1996,** 17, (5-6), 490-519.
